# Application of Physical-Chemical Approaches for Encapsulation of Active Substances in Pharmaceutical and Food Industries

**DOI:** 10.3390/foods12112189

**Published:** 2023-05-30

**Authors:** David Řepka, Antónia Kurillová, Yousef Murtaja, Lubomír Lapčík

**Affiliations:** 1 Department of Physical Chemistry, Faculty of Science, Palacky University Olomouc, 17. Listopadu 12, 771 46 Olomouc, Czech Republic; david.repka01@upol.cz (D.Ř.); antonia.kurillova@upol.cz (A.K.); yousef.murtaja01@upol.cz (Y.M.); 2 Department of Foodstuff Technology, Faculty of Technology, Tomas Bata University in Zlin, Nam. T.G. Masaryka 275, 762 72 Zlin, Czech Republic

**Keywords:** encapsulation, food, pharmaceutical, physico-chemical properties

## Abstract

Background: Encapsulation is a valuable method used to protect active substances and enhance their physico-chemical properties. It can also be used as protection from unpleasant scents and flavors or adverse environmental conditions. Methods: In this comprehensive review, we highlight the methods commonly utilized in the food and pharmaceutical industries, along with recent applications of these methods. Results: Through an analysis of numerous articles published in the last decade, we summarize the key methods and physico-chemical properties that are frequently considered with encapsulation techniques. Conclusion: Encapsulation has demonstrated effectiveness and versatility in multiple industries, such as food, nutraceutical, and pharmaceuticals. Moreover, the selection of appropriate encapsulation methods is critical for the effective encapsulation of specific active compounds. Therefore, constant efforts are being made to develop novel encapsulation methods and coating materials for better encapsulation efficiency and to improve properties for specific use.

## 1. Introduction

**Figure 1:**
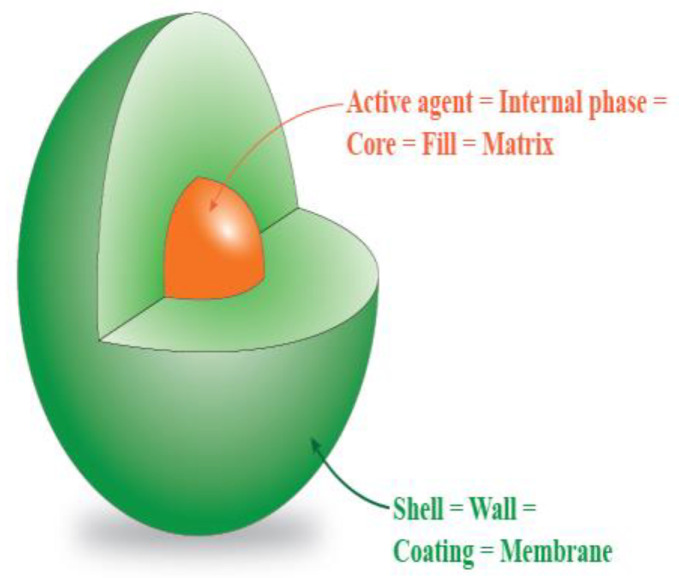
Substance encapsulated into a coating material.

Different substances have great health benefits or function as a drug, but they cannot be used directly or stored for a long period. This is due to their low solubility, low bio-availability, tendency to oxidize, or their strong odor or flavor. Encapsulation is a method that has been known for more than 60 years, but it still holds great interest among researchers [1,2]. Typically, a substance is enclosed by a coating material which forms a barrier to protect the substance from the environment and chemical interaction [3,4,5,6]. Encapsulated substances can be also called core, fill, or matrix substances. The coating material is called a shell or wall material, as can be seen in Figure 1. Encapsulation can be used for improving the mentioned properties of different substances as well as protecting the core substance from, for example, light, moisture, and changes in pH [2,7,8,9]. Apart from protecting of core, it can also be used in the prolongation of release. This can be helpful with gradual and controlled release of drugs [10,11].

Encapsulation can be achieved by many methods, some of which are described in this review. These methods can be divided into three main types: chemical, physico-chemical, and physico-mechanical [7,12]. This review does not mention every encapsulation approach since it is a very broad topic, but rather focuses on methods mostly used in pharmaceutical and food industries. The global food encapsulation market has undergone continuous growth and is expected to reach a value of USD 17 billion by 2027 [13].

With regard to encapsulation of pharmacologically active substances, it is important to note that this technology can have broader applications beyond just the pharmaceutical and food industries. Encapsulation can also be utilized in various other industries; for example, it can be used in nutraceuticals or in the construction industry as a self-healing material such as concrete [14,15]. Specifically, in concrete, types of bacteria can be encapsulated for a self-healing effect [16]. The principle of self-healing materials is applied when the treated material is mechanically damaged; the encapsulate is released after a rupture of capsules and the core material is used to repair the damaged part [17,18,19]. In the same industry, the encapsulation is also used for anticorrosive coating. An example of encapsulated food is linseed oil [20]. The anticorrosive coating can have three mechanisms of protection. Firstly, it is a basic barrier on top of the material; secondly, the coating material can carry corrosion inhibitors; lastly, the coating can provide cathodic protection [21]. Self-healing materials and anticorrosion coatings of materials aim to prolong material use and prevent and avoid structural damage that may occur [22]. For example, marine infrastructure such as bridges and tunnels are in hostile environments and are prone to micro-cracks [23].

Quantum dots (QDs) are closely examined within chemistry. QDs are between 2 and 10 nm in diameter and have a semiconductor core with a shell, together with ligands [24]. Quantum dots can be also used together with encapsulation methods to achieve better properties, which are already unique. Quantum dots are widely used in the biomedical industry [24,25], for example, in biomolecular tracking, tumor imaging, and photodynamic therapy [24]. Encapsulated QDs can be used for the detection of heavy metals and other possible harmful substances [26]. Table 1 provides specific examples of the encapsulation methods described in this review, along with several examples from other industries that demonstrate the diversity of encapsulation.

A retrospective analysis was conducted in this review to evaluate advancements in encapsulation techniques used in the food industry over the last decade. The study identified several methods utilized during this period, including liposomal, lipid, simple and double oil/water emulsions, and extrusion. These techniques have shown significant potential in enhancing food quality and safety by protecting and delivering active ingredients while minimizing the degradation of nutritional and sensory properties.

## 2. Physical-Chemical Approaches in Pharmaceutical Industry

### 2.1. Encapsulation of Bacteriophages

Encapsulation can be used for the treatment of bacterial infections [46]. Due to the still-greater risk of bacteria obtaining resistance to antibiotics, the encapsulation of bacteriophages can be one of the main options to fight bacterial infection without antibiotics [46]. Bacteriophages are viruses that can infect and kill bacteria with high specificity and do not threaten healthy human microflora [46,47,48,49]. Without encapsulation, the bacteriophages have lower survivability in gastro-intestinal conditions; specifically, they can be affected by gastric enzymes, bile, and the acidic environment when used orally [46,48,50].

#### 2.1.1. Emulsification

This method is based on dispersing one liquid with the active compound into a second liquid that is not miscible, and creating small droplets [51,52,53,54,55]. Emulsions tend to be thermodynamically unstable and surfactants as emulsifiers need to be added to decrease surface tension [56,57,58]. Solid particles, such as nanoparticles, can also function as a stabilizer and emulsions stabilized in such a way are called Pickering emulsions [56,58,59].

The method mentioned by Anna Choinska-Pulit et al. includes a mixture of microorganism cells and polymer, which is added to vegetable oil (canola, sunflower, and corn oil, for example) [46]. Mixtures must be homogenous until a water-in-oil emulsion is formed, and stirring of the emulsion is a key step to obtain droplets having the right size and shape. Emulsification creates droplets called capsules, whereas extrusion droplets are called beads. The capsule core is liquid, and the bead core is porous. Capsules are at least 100 times smaller than beads [60].

Emulsifiers, for example, guar gum or lecithin, must be included for stabilization. Settling is used to recover hardened capsules. Researchers in the publication from Dini et al. used this method to encapsulate bacteriophages to reduce enterohemorrhagic *E. coli* in the bovine gastric environment; methoxylated pectin was used as the material for encapsulation and was emulsified by mixing with Tween 20 (Polyoxyethylene sorbitan monolaurate) [61]. Homogenization was performed by mixing and oleic acid was added to reach a final concentration of 10 vol%. Additionally, coating was carried out with 0.2 wt% of high-methoxylated pectin or guar gum.

Double water-in-oil-in-water (W/O/W) emulsion was conducted by Kim, S. et al. [62]. PLGA microspheres were prepared using a bacteriophage solution of pAh-6C in phosphate buffered saline (PBS), mixed with PLGA, and dissolved in dichloromethane to form a W/O emulsion. To form a double emulsion, the primary W/O was homogenized together with polyvinyl alcohol (PVA). A second set of microspheres comprised PLGA/alginate composite. This composite was prepared by preparing W/O/W as before. Calcium chloride solution was added and homogenized to crosslink the alginate. To the emulsion was added deionized water and, after stirring and evaporating, microspheres were obtained [62].

#### 2.1.2. Extrusion

The principle of this method is the forceful flow of material through a slit [8]. Extrusion is a suitable method for the preparation of capsules with hydrocolloids by adding microorganisms. The cell suspension is extruded through a syringe needle in the form of droplets into a bath or a hardening solution, which is mostly calcium chloride [46,63,64,65].

The group of Zhenxing Tang et al. used extrusion as the method to encapsulate Felix O1 bacteriophages into alginate-whey protein microspheres [66]. In this work, researchers focused on the growing problem of bacterial contamination in food poisoning. Worldwide treatment of bacterial infections in food animals is carried out using antibiotics, which causes the overuse of these drugs. Bacteriophage treatment can be a good substitute. Encapsulation is needed because gastric acidity decreases the viability of bacteriophages. Within minutes, the phages were found to be inactive in the simulated gastric fluid. The viability of Felix O1 increased to 2 h of incubation in alginate-whey protein microcapsules [66]. Encapsulation efficiency describes how much of the core material is successfully entrapped into the capsules. The encapsulation efficiency of bacteriophages is calculated as the quantity released from capsules divided by the initial quantity in the capsules multiplied by 100% [67]:

(1) $$EE=\left(\frac{m_{s}}{m_{t}}\right)\cdot 100\%$$

where $m_{i}$ is the initial mass and $m_{s}$ is the mass of the compound left in solution. Here the encapsulation efficiency reached 99% for mixtures of alginate-whey proteins from 93% of pure alginate microspheres. The extrusion method is simple and cheap but has a big disadvantage for use at a mass-production scale [46].

In the publication by Savic et al., researchers encapsulated extracted antioxidants from orange peel into alginate-chitosan microparticles [68]. The extrusion method with coaxial airflow was used for encapsulation. To the solution of alginate 1.5% (*v*/*v*) was added ethanol extract of orange peels. From the homogeneous solution, which was in a plastic syringe, drops were torn using coaxial airflow. Droplets fell into the crosslinking solution while stirring and solidifying. The crosslinking solution was prepared using calcium chloride 2% (*w*/*v*) and chitosan 0.5% (*w*/*v*). The chitosan was prepared using 0.5% (*v*/*v*) acetic acid. The encapsulation efficiency was 89.2% [68].

### 2.2. Probiotic Encapsulation by Chitosan-Gel Particles

Probiotics are helpful bacteria for maintaining a healthy bowel environment. A problem in administering probiotics is that they are prone to degradation because of humidity and low pH in the human intestines. The group of Albadran H. et al. devised a novel method to encapsulate probiotics in chitosan-coated agar-gelatin particles for releasing probiotics into the large intestine [69]. This method should be scalable and thus more suitable than commonly used extrusion methods. Firstly, they prepared agar-gelatin particles loaded with bacteria. This was undertaken by separately dissolving agar and gelatin in deionized water for 2 h at 70–80 °C. Both substances were mixed together at a 1:1 ratio and autoclaved. A small volume of cell suspension was mixed with agar-gelatin and poured into a petri dish, left at room temperature to solidify, and cut into particles having a size of approximately 6 mm. Secondly, particles were coated with chitosan. Agar-gelatin particles were added into the chitosan solution and stirred. Particles were then collected by filtration and washed with phosphate buffered saline (PBS). As a result, particles prepared by the method described by Albadran H. et al. showed great potential for delivery of probiotics into the large intestine [69]. Coated particles showed the ability to withstand the environment of simulated gastric fluid (SGF) for 2 h of incubation and 3 h in simulated intestinal fluid (SIF). X-ray diffraction analysis showed a change in the physico-chemical properties of agar. This change caused by thermal treatment resulted in a strong and tight polymer network.

### 2.3. Nanoemulsion

The definition of a nanoemulsion system can vary as some sources describe that the droplet size is smaller than 500 nm whereas others claim the droplet size is up to 1000 nm [70,71,72,73,74]. The system is made of two immiscible liquids, as stated previously, which are stabilized using surfactant [75,76,77,78]. Nanoemulsions, as opposed to macro and microemulsions, have improved physico-chemical stability [79]. The immiscible liquids used most are water and oil [74,80]. There are two main types of nanoemulsion. The first type can be formed as oil-in-water (O/W); the second type is water-in-oil (W/O), depending on the dispersed liquid. Apart from these, they are also water-in-oil-in-water (W/O/W), and vice versa, and bi-continuous types [55,72,81,82]. Two main approaches are used for preparation, the so-called high and low-energy methods [83,84].

High-energy methods include high-pressure homogenization, microfluidization, and ultrasonication [74,85,86]. With these methods, mechanical energy is used to break large droplets. The main disadvantage of high-energy methods is cost, due to energy demand. Conversely, the advantages are good control of droplet size and possibility of choosing the formulation composition [85,87].

Low-energy methods include the phase inversion temperature and the emulsion inversion point [74,85]. Their principle uses internal chemical energy and formation of droplets with a change in, for example, temperature or chemical composition [86,87,88].

Particles are spheres with amorphous, lipophilic, and negatively charged surfaces [73,89]. A significant number of newly investigated drugs have problems with water solubility and thus their bioavailability is very low [78]. In the study by Dey et al. [90]., it was found that nanoemulsion enhances the absorption of lipids in the small intestine of rats more than conventional emulsion.

The group of Oh et al. [91]. developed a lecithin nano-liposol system loaded with astaxanthin (ASTA). Like other antioxidants, ASTA is susceptible to degradation and thus needs to be encapsulated. The method used for preparation was emulsion evaporation. Chloroform with different concentrations of ASTA was added to lecithin and mixed for 2 h. The final mixture was then added to deionized water and homogenized. Chloroform was removed by drying. The last steps were carried out using ultrasonication and purification by centrifuge. The best results were achieved with a loading of 15 wt% as it had a similar hydrodynamic diameter to that prior of loading. The diameter was around 140 nm ± 4 nm. The encapsulation efficiency for 15 wt% was 98.8% [91].

The publication by Tayeb and Sainsbury describes different uses of nanoemulsion in the pharmaceutical industry [78]. For the delivery of the drug, different ways of application can be used, i.e., nasal, ocular, oral, and parenteral [78,92]. The application of drugs through the skin can be challenging because of the protectiveness of skin layers. Nanoemulsion encapsulation can improve both bioavailability and penetration chance because of the nano-dimensions and low surface tension [78]. Quercetin as a well-known antioxidant with various health benefits, which can be encapsulated. It is necessary to do so because of its poor water solubility, skin absorption, and penetration ability, which are improved by nanoemulsion encapsulation [78,93,94]. Oral drug intake is specific because of the acidic environment and enzymes [95,96]. Encapsulation can decrease the impact of the environment and help with absorption of active substances [97].

## 3. Physical-Chemical Approaches in Food Industry

### 3.1. Spray Drying

The spray-drying process is a common encapsulation method used for the food and pharmaceutical industries due to its cost and ability for use in industrial conditions, for which it has been used since the 1950s [46,98,99,100,101]. The principle of the spray-drying method is the induction of particle agglomeration due to elevated temperatures. Partial melting of the particles and increasing their kinetic energy results in multiple particle collisions [102]. One of the examples of spray-drying encapsulation in the food industry is the encapsulation of oils. This is carried out to protect the oil from oxidization, light, evaporation, and many more effects. One of the side benefits of encapsulating oils or vitamins and antioxidants for supplementation is covering their possible undesirable smell and taste, especially with fish oil [98,103,104,105].

This method is based on three steps. The first is to make small droplets from an emulsion of the core substance with a coating material. This step is called atomization. By producing small droplets, the surface area increases, resulting in faster and easier solvent evaporation [99,106,107,108].

Secondly, this emulsion is then applied and mixed in hot air (100–300 °C) for evaporation of water or another solvent. Liquid is sprayed using various types of nozzles, such as a centrifugal or rotary wheel atomizer, a pressure nozzle, or a two-fluid or pneumatic nozzle [99,100,109].

The last step in spray drying is the separation of dried powder [98,99]. This step is mostly executed by cyclones, which use centrifugal forces to collect particles [100].

Spray drying was used, for example, in encapsulation of flaxseed oil with up to 84% encapsulation efficiency in a study of Fioramonti et al. [110]. In this work, double-layer O/W emulsion was prepared using whey protein concentrate and sodium alginate. The study of Santana Aguiar et al. describes the production of microcapsules using a spray-drying process [111]. Microcapsules loaded with orange essential oil using gelatin and lignin as biopolymers were prepared under optimized conditions. The most important parameters for the atomization efficiency were the inlet air temperature (150 °C), low flow transfer rate (0.15 L/h) of the colloidal suspension, and a higher drying air flow rate (536 L/h). The average particle size was less than 4 μm.

One of the main limitations of spray drying is reduced yield. The reasons for this are associated with the accumulation of powder on the walls during drying. Losing powder through the exhaust air is another reason [7]. Another limitation is due to the high temperature used, and therefore decreased viability can occur for some active substances [46,103,112,113,114]. The encapsulation efficiency, among other things, is affected by the wall material and the material’s physico-chemical properties, such as solubility, viscosity, and thermal stability [104,115].

### 3.2. Freeze Drying

Freeze drying, or lyophilization, is a technique used to retrieve moisture from frozen samples by using sublimation. Sublimation is the phase change when a solid (ice) directly converts to a vapor phase. The process requires heat energy and low pressures for the frozen product to occur. It consists of three main steps: freezing, primary drying, and secondary drying [7,116,117,118]. The first step involves freezing moisture, i.e., crystallization [117]. The product in a vial or a flask is frozen at atmospheric pressure. The result of this phase is the formation of ice crystals. In the second step, the frozen product is placed under a vacuum and the sublimation process takes the main part in primary drying. Lastly, secondary drying is conducted using desorption to remove moisture that was not frozen. Freeze drying is mostly used for food samples and samples with bioactive compounds that are prone to degrading in higher temperatures [7,119,120,121].

The downside of freeze drying is its high operating cost together with a longer process. The freeze-drying process can also cause cell injury, but this can be prevented by using cryoprotectants [7,119,122]. Generally, disaccharides, polyalcohols, amino acids, or proteins function as cryoprotectants [122,123]. To ensure the cryoprotection of the liposomes’ structure, sugar macromolecules (sucrose, lactose, trehalose) are typically included in the liposome systems. Due to the rehydration process, molecules of water are able to replace sugars and reconstitute the liposomes without remarkable changes in their sizes. Sugars such as trehalose are capable of imitating the presence of water and protecting the integrity of dry liposomes and membranes [124].

In a study performed by Li and Deng, a phospholipid t-butyl alcohol water-sucrose solution was initially frozen for 8 h at the temperature T = −40 °C; then, the sample was dried for 48 h at the same temperature, and finally the product was dried at T = 25 °C for 10 h [125]. As a result, the size of the liposomes and polydispersity decreased with the increased concentration of sucrose [124].

The study conducted by Ghasemi et al. focused on encapsulation of orange peel oil [126]. Orange peel oil (OPO) is commonly used as a flavoring in the food industry. Encapsulation is needed because of high volatility and low stability. Pectin solution, whey protein concentrate solution, and maltodextrin solution were mixed to create a biopolymer solution. Tween 80 was added to the solution and mixed until it completely dissolved. OPO was added and homogenized. The resulting solution was created using freeze drying converted into powder. The encapsulation efficiency was in the range from 70% to 88% based on the altered pH of samples. The mean size of particles ranged between 20 and 110 nm.

### 3.3. Encapsulation Using Liposomes

Liposomes are particles made of at least one lipid bilayer shaped into spheres. The bilayer is mostly made of phospholipids and contains hydrophobic and hydrophilic parts [127,128,129,130]. Liposomes, due to their character, can encapsulate substances in both hydrophobic and hydrophilic parts [131,132]. Apart from these two substances, liposomes can be used to encapsulate substances with an amphiphilic character [133,134]. Liposomes are great in drug delivery. High biocompatibility, variability in structural properties, and simple preparation are only a few of the advantages mentioned for the use of liposomes in the biomedical and nutraceutical fields [124]. The group of Hudiyanti, Fawrin, and Siahaan encapsulated vitamin C and beta carotene simultaneously in sesame liposomes [128]. A mixture of beta-carotene and phospholipid was prepared with a ratio of 20% (*w*/*w*). Cholesterol was added to the mixture with the same ratio. For preparation of a thin layer, chloroform was added. Lastly, the vitamin C in the phosphate buffer was added and the mixture was centrifuged. The encapsulation efficiency was different based on the cholesterol used. The highest EE for vitamin C was 89% with 20% (*w*/*w*) of cholesterol used. The highest EE for beta-carotene was 77% without the use of any cholesterol [128].

The researchers Tripathy and Srivastav prepared an extract of *Centella asiatica* leaf encapsulated in liposome [135]. Soy lecithin and stigmasterol were mixed together with ethanol. The solvent was evaporated using a rotary evaporator. To hydrate the newly formed thin lipid film, phosphate buffer solution with the extract was used. Particle sizes ranged from 512.67 to 787.78 nm. The encapsulation efficiency ranged between 40.36 and 67.80% depending on the ratio of used chemicals.

In the work from Mohammadi et al., Spirulina protein hydrolysates were encapsulated [136]. For preparation, lecithin and γ-oryzanol were dissolved in ethanol. With the use of a rotary evaporator, the solvent was removed and a thin phospholipid bilayer was created as the coating of the container. After adding hydrolyzed Spirulina, the liposomes were spontaneously formed. The final size was achieved using a probe sonicator. The encapsulation efficiency reached 90% and the size of particles was approximately 100 nm.

### 3.4. Lipid Encapsulation

**Figure 2:**
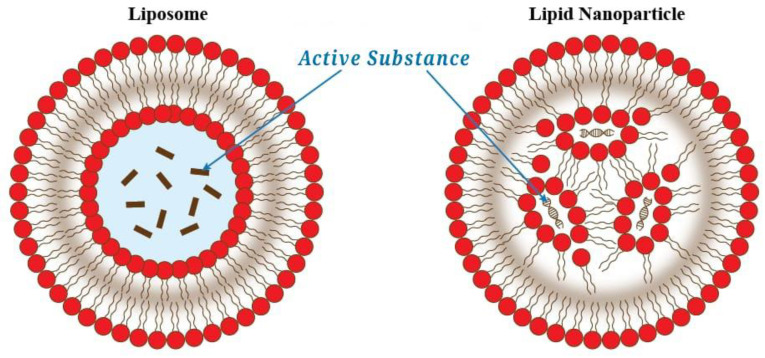
The difference between liposomes and lipid nanoparticles for encapsulation. Redrawn from [137].

Apart from using liposomes as a shell material for encapsulation, lipid nanoparticles can be also used. The main difference can be seen in Figure 2. Liposomes encapsulate active substances in an aqueous environment. By comparison, the lipid nanoparticles do not have a continuous bilayer and the active substance is not encapsulated in an aqueous environment.

Lipids are suitable for encapsulation of drugs and can be used as a drug delivery system [138]. Based on the matrix of lipids they can be separated into different categories. Firstly, if they are composed of only liquid lipids, they are called nanoemulsions. Secondly, those that are made only by solid lipids are called solid lipid nanoparticles (SLNs). Lastly, there can be a crossover of the above. Nanostructured lipid carriers have liquid as well as solid lipids, which are stabilized by the use of surfactants [102,138,139,140].

SLN has some disadvantages, for example, it is prone to gelation and polymorphic transition, and has a low encapsulation efficiency [139]. Based on these disadvantages, the NCL was developed. Physical, chemical, and colloidal stability, as well as the release of the drug, are based on the ratio of solid and liquid lipids in NCL. The ratio can vary from 70:30 in favor of liquid lipids up to 99.9:0.1 [97]. For preparation of SLNs and NCLs, nanoemulsion preparation can be utilized. It is essential to ensure that these procedures are carried out above the melting temperature of the lipid used [141].

Researchers from the group of Lin et al. prepared lipid nanoparticles with entrapped krill oil [142]. Solid lipids were melted with krill oil to form the lipid phase. To form the water phase, distilled water was mixed with lecithin. Both phases were homogenized together and dispersed using an ultrasonic cell crusher. The particle size varied based on the type of solid lipid used. In their work, the scientists achieved a particle size from 112.4 to 989.4 nm. EE was then from 72.86% to 99.37%. The lipid used with the smallest particle size and highest EE was glycerol 1,3-distearate.

### 3.5. Microoemulsion

In the study performed by Ziani et al., the scientists focused on encapsulation of vitamin E, vitamin D, and lemon oil [143]. They used colloidal systems based on surfactants to protect the substances. Emulsion was prepared using 1% (*w*/*w*) of the chosen surfactant and by dissolving it in an acidic buffer solution consisting of 0.8% citric acid and 0.08% sodium benzoate with a final pH of 2.6. A quantity of 10% (*w*/*w*) oil-in-water emulsion was prepared by blending 10% *w*/*w* oil phase with 90% *w*/*w* aqueous phase using a high-speed blender and passing the emulsion through a high-pressure homogenizer [143].

The emulsion titration method was used for preparation of the microemulsion. Emulsion droplets were titrated into aqueous micelle solution. First, the oil solubilized into the surfactants; after a critical concentration of the oil, its droplets remained in solution. In this study it was found that lemon oil was successfully encapsulated into the microemulsion. Both vitamins were not able to form microemulsions [143].

### 3.6. Electrospinning and Electrospraying

Electrospinning and electrospraying are relatively new non-thermal methods producing micro- and nanofibers or particles [144]. These techniques rely on a strong electric field between a polymeric or biopolymeric solution and a grounded collector [145]. One of the primary advantages of these methods is their non-thermal encapsulation, which makes them useful in food and nutraceutical applications [146].

#### 3.6.1. Electrospraying

This method is capable of producing micro/nano thin films, particles, or capsules using a high-voltage electric field [144]. The process is based on atomization while applying an electric force. The solution is pumped into a nozzle where it is further forced by an electric force to create droplets [146]. The surface of the solution is subjected to shear stress because of the high electric potential. As soon as the electrostatic force in the solution is greater than the surface tension, droplets move to the collecting plate [147]. The solvent then evaporates. Electrospraying is used to encapsulate unstable and poorly water-soluble bioactive substances or drugs [148]. Using this method, homogenous nano-sized particles can be formed. It is possible to control particle size by changing the operating parameters; for example, the carrier material has a significant impact on the process [148]. Such a material can be protein-based, which provides aggregation stability, the possibility of different structure forms, and better water dispersibility [148]. Other parameters that have an impact on particle size are electric potential, flow rate, collector distance, viscosity, electrical conductivity, and surface tension [149].

In the study by Mahalakshmi et al., electrospraying was used for encapsulation of curcumin into whey protein [148]. First, they prepared an oil-in-water emulsion. The water phase was achieving by dissolving whey protein in distilled water. For preparation of the oil phase, different quantities of curcumin were dissolved in coconut oil. As an emulsifier in the water phase, Tween 80 was used. Both phases were mixed together to form the final O/W emulsion. The emulsion was sprayed through a needle with the use of electrospraying to obtain nanoparticles. The particles were formed with the use of a 14 kV voltage source at a flow rate of 0.2 mL/h. The particles had a size of less than 500 nm and the electrospraying method achieved 5 to 7% better encapsulation efficiency than the same experiment conducted with conventional spray drying. The encapsulated particles produced using spray drying had a size ranging from 2 to 10 μm [148].

#### 3.6.2. Electrospinning

The electrospinning method produces continuous nanofibers by applying an electrical charge to a polymeric solution. The solution forms a cone on the tip of nozzle because of the electrification. A fluid jet is ejected from the cone toward the grounded collector [150]. Based on different parameters, the size and morphology of fibers can be changed. Apart from this advantage, the electrospinning method manufactures nanofibers that are light, with high porosity and excellent mechanical properties [151]. This method has found application in, for example, food packaging, drug delivery, water treatment, antibacterial and antioxidant materials, catalysis, and wound healing [151,152].

In the study by Pires et al., electrospinning was used for the encapsulation of curcumin into potato starch [153]. The starch solution was mixed with formic acid and shaken for 24 h. Curcumin was added to the starch solution at different dry base concentrations. After different periods of time, the polymer solution was used in electrospraying. The flow of the solution was 0.60 mL/h with an applied +23 kV voltage. The fibers had a mean diameter ranging from 94 nm to 464 nm. The achieved loading capacity was 79.01% to 97.09%.

The advantages and disadvantages of encapsulation methods are summarized in Table 2.

## 4. Future Trends

As previously mentioned, the encapsulation approach holds a promising future in pharmaceutical, food, cosmetics, biomedical, construction, biophysical, and biochemical industries. The most significant advantage would come from developing systems that deliver ingredients precisely where and when they are needed. A controlled and prompt release of the ingredients would also be advantageous. Vitamins, minerals, and other bioactives, which are highly volatile, can be encapsulated to help extend the shelf life and quality of food. In addition, the clever improvement in the sensor-empowered exemplification of medications and prescriptions for constant observation and input of the embodiment frameworks would be a significant advancement, thus enhancing the preparation of the well-being ventures and improving their applicability.

## 5. Conclusions

The process of encapsulation is a highly effective method for preserving and enhancing the physico-chemical properties of active compounds in a range of different applications. These can be, for example, in food, pharmaceutical, nutraceutical, and other industries. In food and pharmaceutical applications, encapsulation has gained more attention in recent years, as evidenced by the increasing number of publications in the Web of Science and the growing market demand. In this review, we focused mainly on food and pharmaceutical industries, where we looked at the methods most commonly used in the past decade. Future research aims are to develop methods with higher encapsulation efficiency that can be easily scaled up for different industries to reduce costs. The selection of an appropriate encapsulation method for a specific group of active compounds is crucial. Therefore, ongoing research is likely to be focused on developing new encapsulation methods and coating materials.

## Figures and Tables

**Table 1:** Different methods of encapsulation.

Methodology	Active Substance	Coating	Vehicle Dimension	Field of Application	Enhanced Property	References
Emulsion electro-spraying assisted by pressurized gas	Algae oil	Wheat gluten extract	3.34 ± 1.77 µm	Nutraceutical industry	Oxidation, bioavailability, organoleptic properties, controlled release	[27]
Extrusion	Polyphenols of *Piper Betel* leaves	Alginate	-	Nutraceutical industry, food supplementation industry	Stability, oxidation, taste	[28]
	Polyphenols from *Mesona chinensis* Benth extract	Alginate	1516.67 ± 40.96 µm	Traditional medicine	Bioaccessibility, bioavailability	[29]
	Phage SL01	Alginate/k-carrageenan	2.110 ± 0.291–2.982 ± 0.477 mm	Pharmaceutical industry	Bioavailability, better survivability (pH, enzymes)	[30]
Nanoemulsion	Thyme oil	Chitosan	50.18 ± 2.32 nm	Bioinsecticides, larvicides	Control release	[31]
	Vitexin	Medium-chain triglyceride	108–166 nm	Food application	Water solubility, bioavailability	[32]
Emulsion	Curcumin	Sunflower oil, carboxy-methylcellu-lose, lecithin	~20 mm	Food delivery	Bioavailability, photochemical stability, less degradation	[33]
Spray drying	Anthocyanins	Maltodextrin	-	Nutraceutical industry, colorant	Shelf life, stability	[34]
	*Saccharomyces boulardii*	Rice protein, maltodextrin	-	Functional foods and beverages, supplements, animal feed	Effectiveness, prolongue storage, less degradation	[35]
Freeze drying	Blackthorn (*Prunus spinosa* L.) extract	Maltodextrin	-	Functional foods, supplements, pharmaceutical	Shelf-life, bioavailability, physico-chemical and biological degradation	[36]
	Propolis	Whey protein isolate	99.76 ± 21.56–242.22 ± 81.78 nm	Alternative medicine, food, cosmetic and pharmaceutical industries	Odor, taste, bioavailability	[37,38]
Lipid encapsulation	Gamma-oryzanol	Stearic acid, sunflower oil/rice bran phospholipids, Tween 80	143 ± 3.46 nm	Nutraceutical industry	Water solubility, size	[39]
Internal phase separation	N-acetylcysteine	Ethylcellulose	100–1000 µm	Nutritional supplement	Bitter aftertaste, astringency, sulfur smell	[40]
Self-assembly of biopolymers	Anthocyanins	Whey protein isolate, pectin	~ 200 nm	Nutraceutical industry, colorant	Molecular instability	[41]
Vacuum facilitated infusion	Curcumin	*Geotrichum candidum arthrospores*	-	Food industry, pharmaceutical industry	Water solubility, chemical stability, Bioavailability	[42]
Multiple step preparation including modified Störber sol-gel process	Mn_3_O_4_	Hollow carbon sphere coated by graphene layer	-	Battery industry	Enhancing performance of lithium-ion batteries, specific capacity	[43]
Synthesis of QD, growth of iron shell, and oxidation to form iron oxide shell	Quantum dots	Iron oxide	~20 nm	Bifunctional markers, virus detection	Optical properties	[44]
Pelletization process, coating processes	Calcium acetate/sodium carbonate (or composite of two), superabsorbent polymers, poly(ethylene glycol)	Epoxy resin, fine sand	-	Self-healing concrete	Waterproof and alkali resistance, mineralization time, durability	[14]
Sol-gel method	SiO_2_	ZnO	-	Cosmetics, renewable energy, UV-protecting	Photoactivity properties	[45]

**Table 2:** Advantages and disadvantages of encapsulation methods.

Method	Advantages	Disadvantages	References
Extrusion	Cost-effective, high production capacity, no organic solvents or high temperatures	Difficult scaling up, lower entrapment, problematic with viscous solutions, larger particles,	[46,119,154]
Freeze drying	Highly porous material, higher entrapment, low operating temperature	High cost, time consuming, possible cell injury	[144,154,155]
Spray drying	Large scale method, continuous, flexibility, rapid use, high efficiency, low cost	Possible thermal degradation, poor size control	[119,155,156,157]
Lipid encapsulation	Scalable, biodegradability, biocompatibility, controlled release	SLNs—low drug loading, possible gelation, drug expulsion during storage	[158,159]
Microemulsion	Scalable, easy preparation, low cost	low stability, poor delivery properties	[144,157,160]
Nanoemulsion	Kinetic stability, better optical properties	Rapid-release	[160,161]
Encapsulation using liposomes	Controlled release, greater stability, high bioavailability, biocompatibility, and biodegradability	High cost, low physico-chemical stability, variation in particle size	[154]
Electrospinning/electrospraying	No thermal degradation, possible size control, simplicity, low starting cost	Not yet in larger scale, specific equipment	[144,148,151,152,162,163]

## Data Availability

The data presented in this study are available on request from the corresponding author.
